# Large-scale genomic analysis reveals significant role of insertion sequences in antimicrobial resistance of *Acinetobacter baumannii*

**DOI:** 10.1128/mbio.02852-24

**Published:** 2025-02-20

**Authors:** Fei Xie, Lifeng Wang, Song Li, Long Hu, Yanhua Wen, Xuming Li, Kun Ye, Zhimei Duan, Qi Wang, Yuanlin Guan, Ye Zhang, Qiqi Shi, Jiyong Yang, Han Xia, Lixin Xie

**Affiliations:** 1 College of Pulmonary and Critical Care Medicine, Chinese PLA General Hospital, Beijing, China; 2 Laboratory Medicine Department, First Medical Center of Chinese PLA General Hospital, Beijing, China; 3 Department of Research and Development, Hugobiotech Co., Ltd, Beijing, China; University of Pretoria, Pretoria, Gauteng, South Africa

**Keywords:** antimicrobial resistance, random-forest, insertion sequence, IS-ARG pairs

## Abstract

*Acinetobacter baumannii*, a prominent nosocomial pathogen renowned for its extensive resistance to antimicrobial agents, poses a significant challenge in the accurate prediction of antimicrobial resistance (AMR) from genomic data. Despite thorough researches on the molecular mechanisms of AMR, gaps remain in our understanding of key contributors. This study utilized rule-based and three machine learning models to predict AMR phenotypes, aiming to decipher key genomic factors associated with AMR. Genomes and antibiotic resistance phenotypes from 1,012 public isolates were employed for model construction and training. To validate the models, a data set comprising 164 self-collected strains underwent next-generation sequencing, nanopore long-read sequencing, and antimicrobial susceptibility testing using the broth dilution method. It was found that the presence of antibiotic resistance genes (ARGs) alone was insufficient to accurately predict AMR phenotype for the majority of antibiotics (90%, 18 out of 20) in the public data set. Conversely, it was observed that combining ARGs with insertion sequence (IS) elements significantly enhanced predictive performance. The Random Forest model was found to outperform the support vector machine (SVM), logistic regression model, and rule-based method across all 20 antibiotics, with accuracies ranging from 83.80% to 97.70%. In the validation data set, even higher accuracies were achieved, ranging from 85.63% to 99.31%. Furthermore, conserved sequence patterns between IS elements and ARGs were validated using self-collected long-read sequencing data, substantially enhancing the accuracy of AMR prediction in *A. baumannii*. This study underscores the pivotal role of IS elements in AMR.

**IMPORTANCE:**

The interplay between insertion sequences (ISs) and antibiotic resistance genes (ARGs) in *Acinetobacter baumannii* contributes to resistance against specific antibiotics. Conventionally, genetic variations and ARGs have been utilized for predicting resistance phenotypes, with the potential pivotal role of IS elements largely overlooked. Our study advances this approach by integrating both rule-based and machine learning models to predict AMR in *A. baumannii*. This significantly enhances the accuracy of AMR prediction, emphasizing the pivotal function of IS elements in antibiotic resistance. Notably, we uncover a series of conserved sequence patterns linking IS elements and ARGs, which outperform ARGs alone in phenotypic prediction. Our findings are crucial for bioinformatics strategies aimed at studying and tracking AMR, offering novel insights into combating the escalating AMR challenge.

## INTRODUCTION

Antimicrobial resistance (AMR) in bacterial pathogens is rapidly increasing globally and is associated with high morbidity and mortality rates (1). Multidrug-resistant (MDR) gram-negative bacterial pathogens are difficult to treat and may even be untreatable with conventional antibiotics, which is a growing and pressing global health concern (2, 3). *Acinetobacter baumannii* is an important nosocomial pathogen that has emerged as a cause of numerous global outbreaks and has been reported to cause serious health problems (4–6). *A. baumannii* has an extraordinary capacity to develop resistance to multiple antimicrobial agents by acquiring antibiotic resistance genes (ARGs) (7). In addition to efforts to develop new drugs, there is an urgent need for preclinical tools that can effectively and rapidly detect AMR to address infections caused by MDR *A. baumannii* (8).

Antimicrobial susceptibility testing (AST) currently serves as the primary method for identifying antimicrobial phenotypes in clinical pathogens. Nevertheless, this approach has many limitations, such as being only viable for cultured strains, requiring microbiology facilities, trained clinical microbial person for accuracy, and being time-consuming (9). Recent advancements in whole genome sequencing are expanding our ability to detect and study AMR (10). Sequencing-based methods and resources such as CARD (11), Resfinder (12), and PATRIC (13) have revolutionized AMR research, promising genomics-based surveillance, and prediction of resistance phenotype (14). For some important pathogens, genomic features with good concordance to resistance phenotypes have been identified, such as gentamicin (GEN) in *Escherichia coli* (15) and AMK in *Mycobacterium tuberculosis* (16). However, for other pathogens, the rule-based approaches cannot be directly employed to detect AMR due to an incomplete understanding of the underlying resistant mechanisms (17). Previous studies have attempted to capture uncertainty in the genetic basis of resistance, and machine learning algorithms have been extensively employed to construct models that predict AMR by directly learning valuable information from the data (18, 19). Random forest (RF)-based approach has been proven to be an effective method for predicting AMR phenotypes (20, 21). However, many of these methods have ignored the potential roles of insertion sequences (ISs) in the prediction of AMR.

IS elements, with sizes typically less than 2.5 kb, represent the smallest and the most abundant transposable elements. They play a crucial role in genomic plasticity and evolution in microbial genomes (22). They influence the resistance phenotype of host bacteria through multiple mechanisms. Firstly, as components of composite transposons, IS elements, while traditionally not carrying “passenger” genes themselves, can mobilize regions containing resistance genes, forming structures defined by two identical or related IS elements, which enable the spread of resistance genes between different DNA molecules (23). Secondly, IS elements can directly carry and mobilize one or more resistance genes within their adjacent regions, especially in gram-negative bacteria, often containing strong promoters that drive the expression of captured genes (24). Thirdly, IS elements can alter gene expression through insertion by modifying upstream sequences of genes, impacting gene expression levels (25, 26). These mechanisms are prevalent among ESKAPE pathogens (*Enterococcus faecium*, *Staphylococcus aureus*, *Klebsiella pneumoniae*, *Acinetobacter baumannii*, *Pseudomonas aeruginosa*, and *Enterobacter* species) and collectively facilitate the horizontal transfer of ARGs between bacteria, accelerating the emergence and spread of MDR bacteria and posing a significant threat to global public health (27, 28). In *A. baumannii*, the interactions between IS elements and ARGs have been reported to be responsible for the resistance to certain antibiotics. For example, *A. baumannii* isolates with IS*Aba1-bla*_OXA-23_ were highly resistant to carbapenems (29, 30). The presence of IS*Aba11* in either the *lpxA* or *lpxC* gene can result in the complete loss of lipopolysaccharide production and a high level of colistin (COL) resistance (31). Therefore, IS elements may provide key predictive power for the AMR of *A. baumannii*.

In this study, we developed RF-based models to predict AMR and systematically evaluated the predictive performance using 1,012 public *A. baumannii* isolates. Additionally, we compared the consistency of the key features identified by rule-based method and phenotypic prediction model for each antibiotic and explored the associations between IS-ARG pairs and resistance phenotypes. For further validation, we assembled 164 high-quality genomes of clinical *A. baumannii* isolates by combing Illumina short reads and nanopore long reads. AST for these isolates was performed using broth microdilution assay method. The predictive effectiveness of the phenotypic prediction model and the potential patterns through which IS elements could affect AMR were confirmed in the self-collected data set. Together, we developed RF-based model that highly improved the accuracy of predicting AMR phenotypes and universally investigate the impacts of IS-ARG relationships on resistance phenotypes in *A. baumannii*.

## RESULTS

### The public PATRIC data set used for AMR prediction

**Fig 1:**
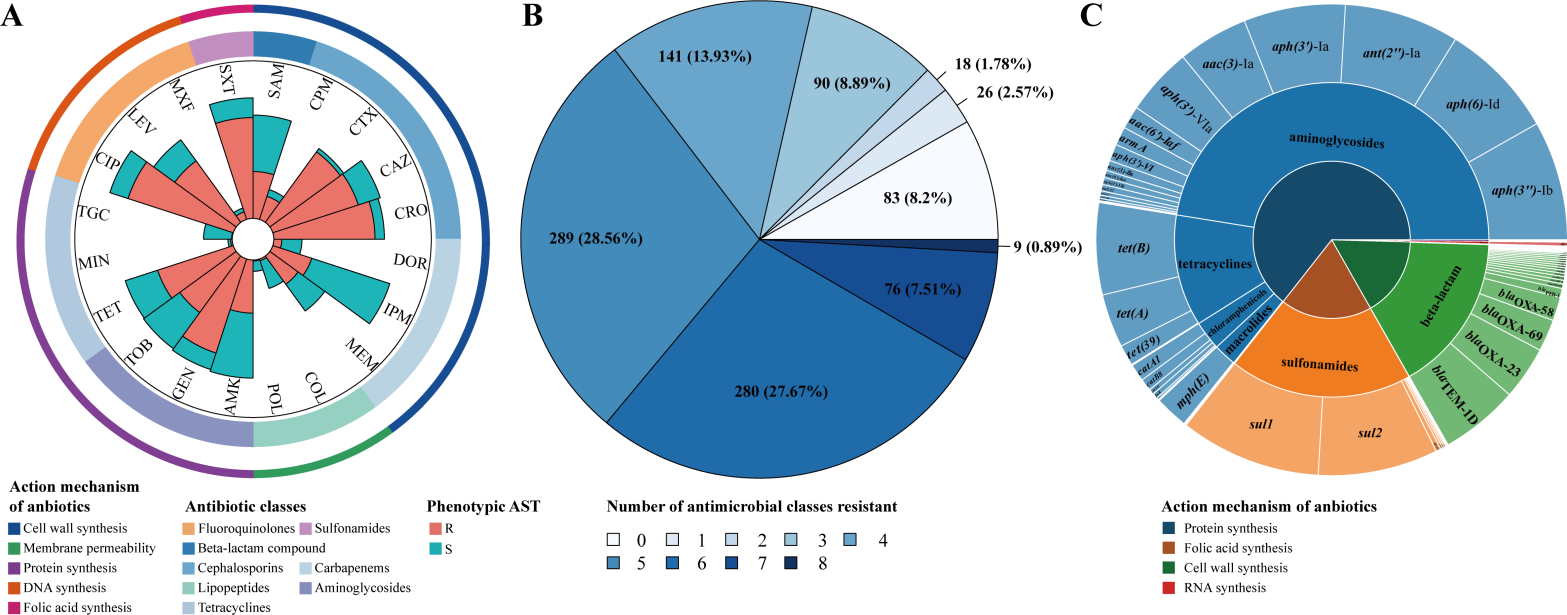
Resistance phenotype and genotype of public genomes. (A) Antimicrobial susceptibility testing profiles against the 20 antibiotics. The outer circle denotes the action mechanism of drug, and the inner circle stands for the antibiotic type. Barplot denotes the number of resistant (R, red) and sensitive (S, green) samples. (**B**) Proportion of resistant samples. A color-coded representation was employed to visualize the results, where darker shades indicated a higher number of resistant categories. A value of 0 represented susceptibility to all eight classes of antibiotics, while a value of 8 indicated resistance to all eight classes antibiotics. (**C**) Sunburst diagram detailing the distribution of AMR genes from Resfinder database. The inner circle represents the action mechanism of drugs, the middle circle represents the antibiotic type, and the outer circle represents the number of samples carrying the ARGs.

Initially, we employed rule-based strategy to identify the key factors responsible for resistance phenotypes in 1,012 public isolates. A total of 1,012 *A*. *baumannii* obtained from the Pathosystems Resource Integration Center (PATRIC; https://patricbrc.org/) were enrolled in this study (Table S1). These strains were collected from 1999 to 2019, with the highest number of isolates in 2004 (21.25%, 215 out of 1,012), followed by 2005 (13.54%, 137 out of 1,012) (Fig. S1). A total of 20 antibiotics belonging to eight classes were studied, including beta-lactam compound (5%, 1), cephalosporins (20%, 4), carbapenems (15%, 3), lipopeptides (10%, 2), aminoglycosides (15%, 3), tetracyclines (15%, 3), fluoroquinolones (15%, 3), and sulfonamide (5%, 1) (Fig. 1A and Table 1). Among these 1,012 strains, a total of 885 strains exhibited MDR phenotype, indicating resistance to three or more classes of antibiotics. Notably, nine strains were found to be resistant to all eight classes of antibiotics. Among the remaining 127 strains, 83 were susceptible to all eight classes of antibiotics (Fig. 1B). A total of 5,622 ARGs were identified among the 1,012 strains. Aminoglycoside resistance genes were the most prevalent, accounting for 47.6% (2,675). Sulfonamide, beta-lactamase, and tetracycline resistance genes also exceeded 10%, representing 18.8%, 16.3%, and 11.5% of the total, respectively (Fig. 1C). The ARGs *sul1* (546), *sul2* (464), and *aph(3'')-*Ib (462) were the three most prevalent genes, and the carbapenem resistance gene *bla*_OXA-23_ was identified in 206 (20.36%) isolates.

**TABLE 1:** Statistics of the antibiotic resistance of 1,012 public *A. baumannii* isolates

Antibiotic	Antibiotic classes	Action mechanism of antibiotics	Number of resistant isolates	Number of sensitive isolates
SAM	Beta-lactam compound	Cell wall synthesis	357	430
CPM	Cephalosporins	Cell wall synthesis	185	54
CTX	Cephalosporins	Cell wall synthesis	664	31
CAZ	Cephalosporins	Cell wall synthesis	685	176
CRO	Cephalosporins	Cell wall synthesis	771	72
DOR	Carbapenems	Cell wall synthesis	61	154
IPM	Carbapenems	Cell wall synthesis	312	626
MEM	Carbapenems	Cell wall synthesis	266	252
COL	Lipopeptides	Membrane permeability	14	222
POL	Lipopeptides	Membrane permeability	8	79
AMK	Aminoglycosides	Protein synthesis	405	495
GEN	Aminoglycosides	Protein synthesis	754	132
TOB	Aminoglycosides	Protein synthesis	532	341
TET	Tetracyclines	Protein synthesis	606	259
MIN	Tetracyclines	Protein synthesis	12	21
TGC	Tetracyclines	Protein synthesis	5	214
CIP	Fluoroquinolones	DNA synthesis	843	146
LEV	Fluoroquinolones	DNA synthesis	579	198
MXF	Fluoroquinolones	DNA synthesis	60	31
SXT	Sulfonamides	Folic acid synthesis	768	150

### RF-based model outperformed in AMR prediction

**Fig 2:**
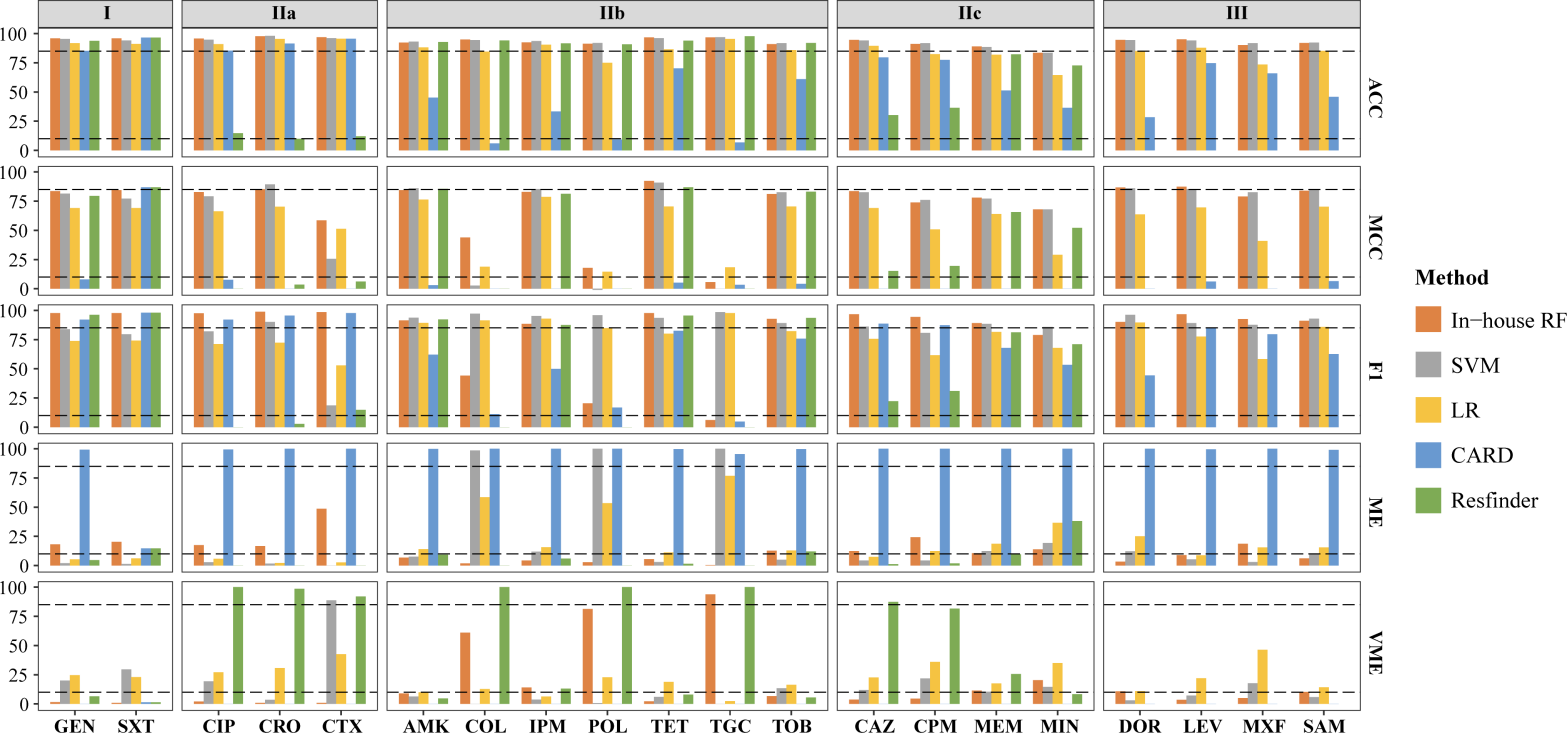
Comparison of performances between rule-based methods and machine learning methods. Accuracy (ACC), Matthew’s correlation coefficient (MCC), F1-score, very major error (VME) rate, and major error (ME) rate were used. For the metrics of ACC, MCC, and F1-score, the dashed line represents 80%. For ME and VME, the dashed line represents 10%.

The phenotypic data, which combined genomic sequences of the strains obtained from PATRIC database, were utilized to select the key feature sets and construct the phenotypic prediction model for each drug. Unfortunately, the rule-based methods yielded disappointed results. Out of the 20 antibiotics, only two (GEN and trimethoprim-sulfamethoxazole [SXT]) demonstrated high prediction effectiveness in both CARD and Resfinder databases. Ten antibiotics exhibited desirable accuracy in a single method, and the remaining eight antibiotics failed to achieve acceptable results with either method. Therefore, we constructed machine learning models to improve the prediction (Fig. S2). Our in-house model demonstrated excellent performance in all 20 antibiotics, with the accuracy (ACC) ranging from 83.63% to 97.68% (Fig. S3). Only three antibiotics (COL, polymyxin B [POL], and tigecycline [TGC]) exhibited low Matthews correlation coefficient (MCC) and F1 scores due to a significant imbalance in the numbers of drug-resistant and drug-sensitive isolates. Compared with the rule-based methods, our phenotypic prediction model not only achieved varying degrees of improvement in accuracy but also demonstrated advantages in the terms of major error (ME) rate, very ME (VME), MCC, and F1 (Fig. 2). The RF-based model achieved an average accuracy of 93.40% in predicting 20 antibiotic resistance phenotypes, while the support vector machine (SVM) and logistic regression (LR) models achieved accuracies of 93.35% and 86.05%, respectively. Additionally, the RF model had lower ME and VME compared to the other two models (ME: RF 12.68%, SVM 20.23%, LR 20.20%; VME rate: RF 17.17%, SVM 14.17%, LR: 22.09%) (Table S2). Overall, the RF based model outperformed the SVM and LR models in AMR phenotype prediction.

Overall, the 20 drugs can be divided into three main groups according to the predictive accuracy of rule-based approaches in CARD and Resfinder databases. The group I included two drugs (GEN and SXT) that exhibited good performances in both databases. Fourteen drugs with at least one poor result were classified as group II, which could be further divided into three subgroups: IIa (ciprofloxacin [CIP], ceftriaxone [CRO], cefotaxime [CTX]) with poor performance in Resfinder, IIb (AMK], COL, imipenem [IPM], POL, tetracycline [TET], TGC, and tobramycin [TOB]) with poor performance in CARD and IIc (ceftazidime [CAZ], cefepime [CPM], meropenem [MEM], and minocycline [MIN]) with poor performance in both databases. Group III consisted of four drugs (doripenem [DOR], levofloxacin [LEV], moxifloxacin [MXF], and ampicillin-sulbactam [SAM]) without Resfinder result. For the two drugs in group I, although the accuracies were similar in all models, the rule-based methods performed poorly in ME and VME. For drugs in group IIa, the accuracies of the rule-based method in CARD were approximately 85%, while in Resfinder, they were only about 10%. However, the RF-based models achieved an overall accuracy above 90%. VME rates in Resfinder exceeded 75% for the three drugs of group IIa, while the RF-based models exhibited low levels. For drugs in group IIb, the performances of rule-based methods in Resfinder were comparable to RF-based models, while they were poor in CARD. Therefore, the choice of database was crucial when applying a rule-based approach to predict AMR. For both group IIc and group III, the prediction accuracies of models improved significantly, and ME and VME rates were greatly reduced compared to the rule-based methods. In summary, these results highlighted the versatility and robustness of the RF-based model.

### IS elements acting as key factors in AMR prediction

**Fig 3:**
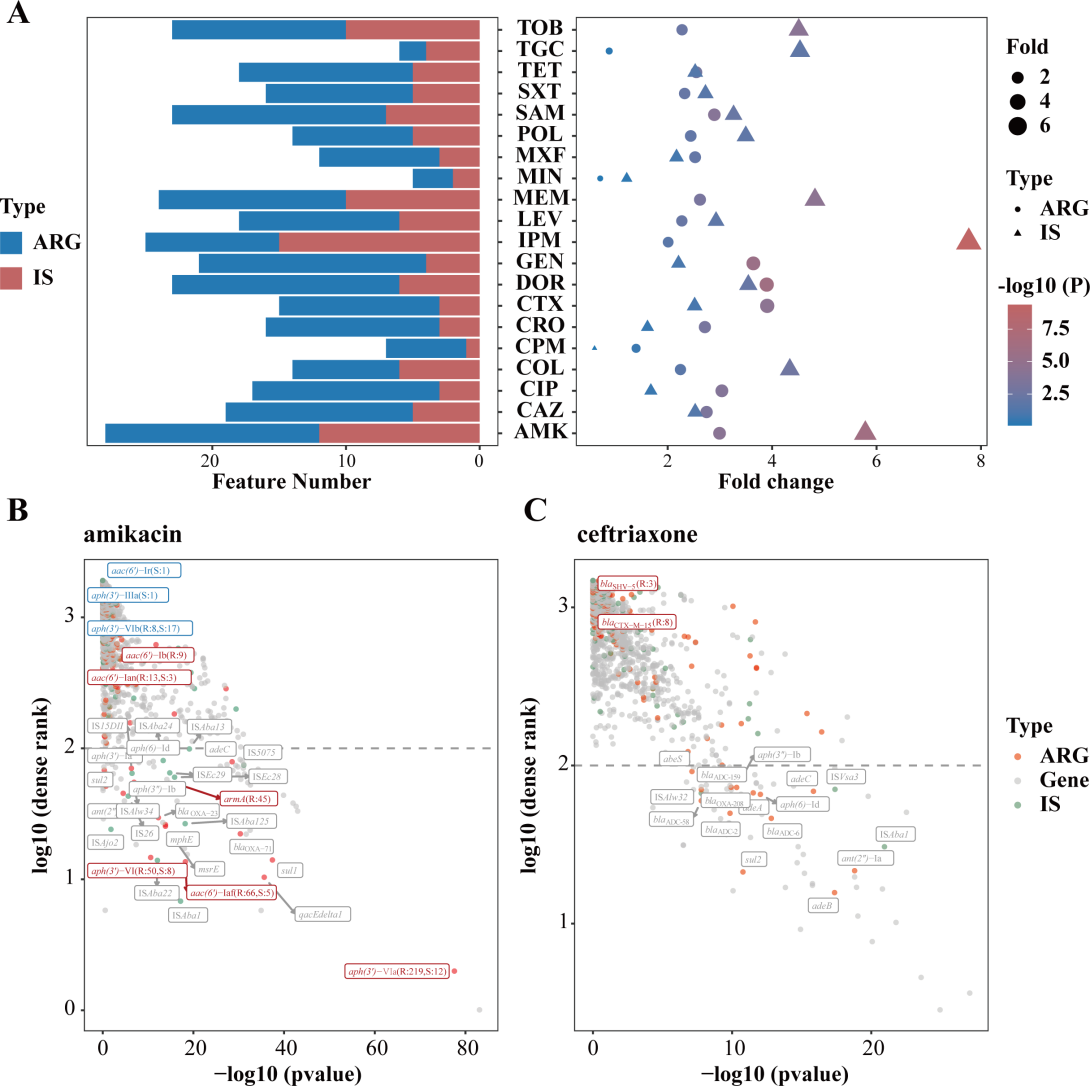
Key features identified based on RF-based model. (**A**) Distribution of ARGs and IS elements in the key feature sets of 20 drugs. The left figure shows the number of IS elements and ARGs. The right figure is the result of enrichment analysis of ARGs and IS elements in key feature sets by hypergeometric testing in different drugs. (**B, C**) Comparison of features determined by RF model with contributed features from Resfinder database for AMK and CRO. Scatter plot is used to show the feature significance (Fisher’s exact test between R and S, *x*-axis) and dense rank (*y*-axis) for all features in RF model. ARGs are highlighted as red dots, IS elements are highlighted as green dots, and other prokka genes are black dots. All IS elements and ARGs of which dense rank less than 100 (gray dashed line) and ARGs contributed to the drug in Resfinder are labeled. Resfinder ARGs of which strains number of R more than S are labeled red, otherwise blue.

Compared to the rule-based models, the RF-based model identified a considerable number of IS elements as key features, in addition to ARGs. The lists of key features showed that ARGs and IS elements accounted for a significant proportion of most antibiotics (Fig. 3A). For the majority of drugs (90%, 18 out of 20), the number of ARGs was greater than that of IS elements in the key feature sets, with the exceptions of IPM and TGC. A total of 5,159 genetic elements were identified in these strains, of which 6.14% were ARGs and 2.38% were IS elements. Enrichment analysis showed that ARGs and IS elements were significantly enriched in key feature sets of 17 drugs and 13 drugs, respectively (average fold change ≥ 2 and *P* ≤ 0.05). These results indicated that IS elements may be as important as ARGs for resistance phenotypes of *A. baumannii*.

We further compared the consistency of key features for each antibiotic between rule-based method and RF-based model. In most cases, the key features identified by these two methods were not identical, and even when the same features were identified, they had different rankings of importance. Some key features identified by the rule-based model were absent in the RF-based model and vice versa. For example, in the case of AMK, which showed good performance in Resfinder, the rule-based method identifies *aph(3′)-*VIa as the most significantly important ARG, while it ranked second in the RF model. Additionally, two ARGs (*aac(6′)-*Ib and *aac(6*′)-Ian) that were not significant enough and three ARGs [*aac(6*′)-Ir, *aph(3*′)-IIIa, and *aph(3*′)-VIb] that potentially interfere with predictions in rule-based method were excluded from key feature sets of RF-based model (Fig. 3B). In the case of CRO, which had poor performance in Resfinder, none of ARGs were identified as key features in rule-based methods. On the contrary, RF-based model identified more features, including three beta-lactamase genes (*bla*_ADC-2_, *bla*_ADC-6_, and *bla*_OXA-258_), three aminoglycoside genes [*aph(6*′)-Id, *aph(3*″)-IIb, and *ant(2*″)-IIa], two effllux pump-related genes (*adeA* and *adeC*), and two IS elements (IS*Alw32* and IS*Aba1*) (Fig. 3C).

### IS-ARG pairs are conservative and strongly linked to AMR

**Fig 4:**
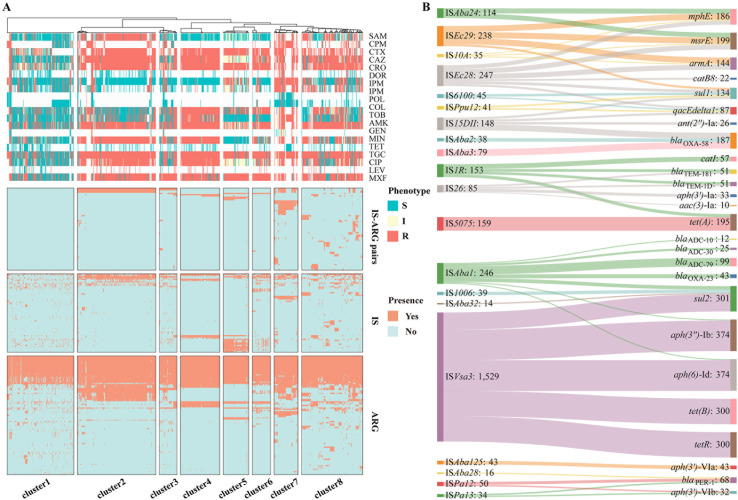
IS-ARG pairs distribution in 1,012 public *A. baumannii* isolates. (**A**) The landscape of ARGs, IS elements, and IS-ARG features in all samples. Unsupervised bidirectional clustering was performed based on IS-ARG features and samples were divided into eight clusters. The top bars above the heatmap show the antibiotics sensitivity for 20 drugs, respectively. (**B**) Sankey diagram showing the prevalence of different IS elements (left) detected closely to the ARGs (right). The height of axis corresponds to the sum of samples number of element linked to ARGs or IS elements.

IS elements are generally considered to play an important role in the evolution and plasticity of bacterial genome, and studies have reported that some IS elements can co-locate with ARGs, making them an inherent risk factor for AMR (23, 32). Therefore, we investigated the association of relationships between IS elements and ARGs with resistance phenotypes. Using bidirectional unsupervised clustering based on IS-ARG pairs identified across whole genomes, the strains were clustered into eight groups, each exhibiting different patterns of resistance phenotypes (Fig. 4A). For isolates in cluster 1, although IS elements and ARGs were abundantly distributed in their genomes, IS-ARG pairs were missing. Coincidentally, these isolates were sensitive to almost all of 20 drugs. This suggested that the presence of ARG alone was not sufficient to confer antibiotic resistance, and the absence of some specific IS-ARG pairs may be a key factor for sensitivity phenotypes. In other clusters, the presence of specific IS-ARG pairs directly corresponded to MDR phenotypes. For example, in cluster 2, several IS-ARG pairs, including IS*Vsa3-aph(3″)-*Ib, IS*Vsa3-aph(6)*-Id , IS*Vsa3-tet(B*), and IS*Vsa3-tetR*, were prevalent. The presence of these IS-ARG pairs perfectly matched the resistance phenotypes of various drugs (SAM, CAZ, CTX, CRO, GEN, TET, CIP, LEV, and SXT). This phenomenon was also observed in other clusters, such as IS*Aba1-bla*_ADC_ and IS*5075-tet(A*) linked to resistance of 10 antibiotics (CTX, CAZ, CRO, TOB, AMK, GEN, TET, CIP, LEV, SXT) in cluster 4, and IS*Vsa3-sul2*, IS*Aba3-bla*_OXA-58_, IS*15DII-bla*_OXA-58_, and IS*Aba2-bla*_OXA-58_ tied to resistance of four drugs (CTX, CRO, CIP, and SXT) in cluster 5. In summary, the presences of specific IS-ARG pairs were directly correlated with resistance phenotypes of certain antibiotics.

Then, we conducted a comprehensive analysis of the distribution characteristics of IS-ARG pairs and their interaction patterns in these 1,012 public isolates (Fig. 4B). We observed plentiful co-existence of IS elements with specific ARGs, and vice versa. Notably, IS elements were found to form stable interactions with multiple ARGs in a higher frequency (14/21, 66.67%) (Table S3). For example, IS*Vsa3* was frequently associated with five ARGs belonging to three different classes [aminoglycosides: *aph(3*″*)*-Ib, *aph(6)*-Id; tetracycline: *tet(B)* and *tetR*; and sulfonamides: *sul2*], while IS*5075* specifically interacted with *tet(A*). Conversely, a majority of the ARGs (15/27, 55.56%) were also observed to be correlated with multiple IS elements. For instance, *aac(3)*-Ia and *aph(3*′*)*-Ia were specifically correlated with IS*26*, and *bla*_ADC-10_, *bla*_ADC-30_, *bla*_ADC-79_, and *bla*_OXA-23_ were specifically correlated with IS*Aba1*. Furthermore, a bidirectional conservative IS-ARG pair [IS*Aba125-aph(3*′*)*-VIa] was also identified. The effects of certain IS-ARG pairs on the AMR phenotype in *A. baumannii* have been verified in previous studies, such as IS*Aba1* forming the hybrid promoter that affecting the carbapenem resistance (33, 34), or IS*26* driving replication and increasing expressions of ARGs, leading to AMR (35, 36). Overall, IS elements play a significant role in AMR through various mechanisms, primarily involving the recruitment, transfer, and cooperation with ARGs.

### Validation with 164 self-collected *A. baumannii* isolates

**Fig 5:**
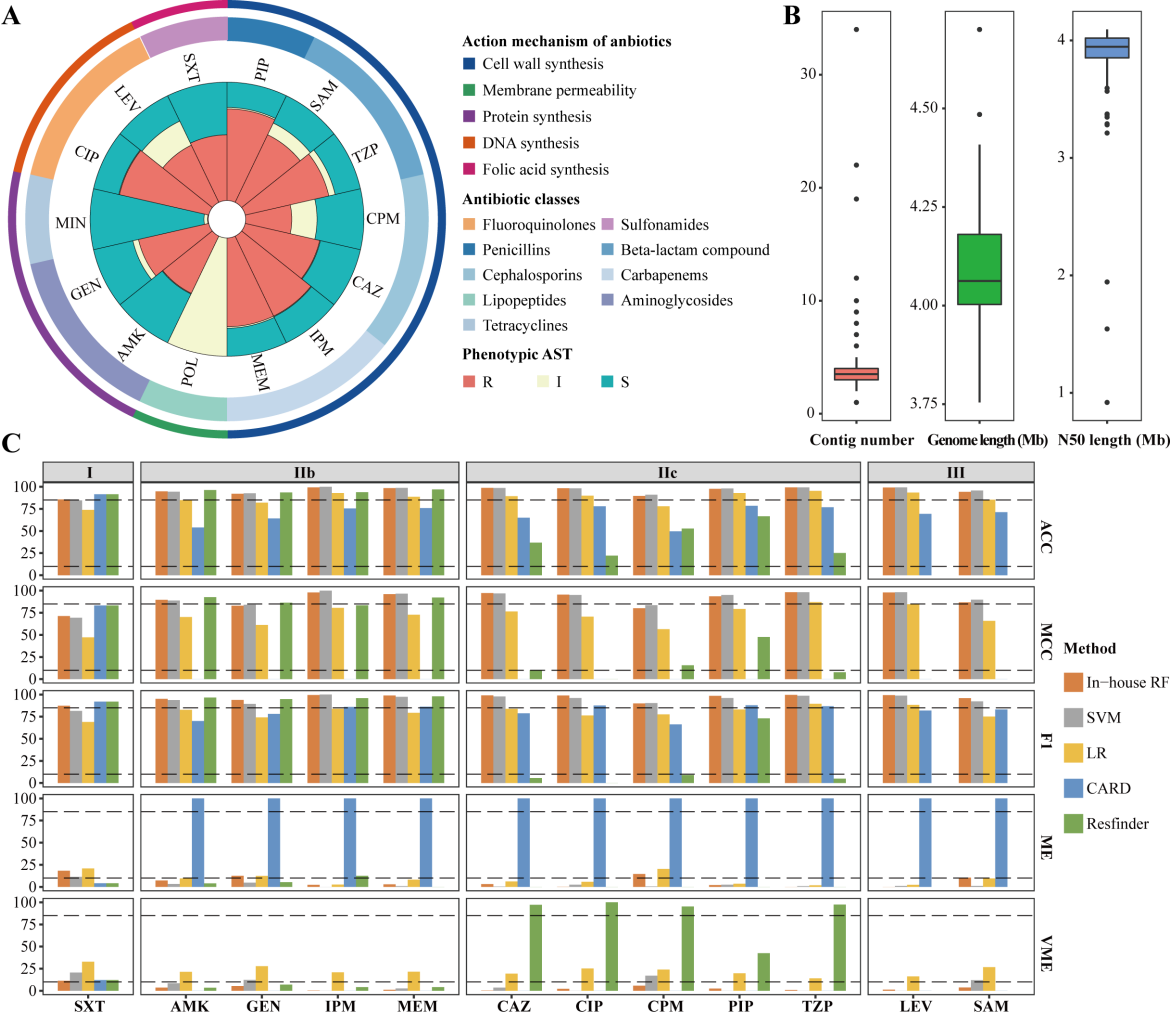
Antimicrobial susceptibility profiles, genome evaluation, and performance of machine learning models in self-collected *A. baumannii* isolates. (**A**) Sunburst diagram representing the AMR phenotypes of 164 self-collected *A. baumannii* isolates against 12 antibiotics categorized into nine classes. The circles from outer to inner represent the mechanism of action of the antibiotics, the antibiotic classes, and the resistance phenotypes, respectively. (**B**) Distribution of various quality parameters for clinically isolated genomes. (**C**) Comparison of performance among rule-based methods, SVM, LR, and in-house RF model.

To validate the aforementioned findings, an independent cohort consisting of 164 *A*. *baumannii* isolates was analyzed. These isolates were collected from 164 patients admitted to the Chinese People’s Liberation Army General Hospital between January 2018 and December 2020, originating from various types of specimens such as sputum, blood, and bronchoalveolar lavage fluid. ASTs were performed for 14 antibiotics, with MIN and POL excluded from subsequent analysis due to the low number of resistant or sensitive isoates (fewer than five) (Fig. 5A; Table 2; Table S4). Despite the identification of many IS-ARG pairs in the public data, IS-ARG pairs located at the terminals of contigs might be ignored due to the poor genome qualities (Fig. S4 and S5). To address this limitation, we utilized a combination of NGS short reads and nanopore long reads to construct high-quality genomes of 164 isolates. The results showed high-quality assemblies with N50 values exceeding 3 Mb for the majority of isolates (98.17%, 161/164) and a maximum of 10 contigs for most isolates (96.95%, 159/164) (Fig. 5B and Table S5). These high-quality data were employed to evaluate rule-based and RF-based models. Overall, RF-based models exhibited prediction accuracies ranging from 85.62% to 99.31% (Fig. S6 and Table S6). In the data set of 164 self-collected *A. baumannii* isolates, the average accuracies of the RF, SVM, and LR models in predicting antibiotic resistance phenotypes were 95.59%, 95.85%, and 87.19%, respectively. The average ME rates were 6.13% for RF, 2.41% for SVM, and 8.56% for LR. The average VME rates were 3.19% for RF, 6.36% for SVM, and 22.48% for LR. From these results, the performance of the RF and SVM models was comparable in terms of accuracy. However, VME is generally considered a more serious type of prediction error than ME. Taking this into account, the RF model still outperformed the other models overall.

**TABLE 2:** Statistics of the antibiotic resistance of 164 self-collected *A. baumannii* isolates

Antibiotic	Antibiotic classes	Action mechanism of antibiotics	Number of resistant isolates	Number of sensitive isolates	Number of intermediate isolates
PIP	Penicillins	Cell wall synthesis	127	35	2
SAM	Beta-lactam compound	Cell wall synthesis	104	42	18
TZP	Beta-lactam compound	Cell wall synthesis	119	36	9
CPM	Cephalosporins	Cell wall synthesis	64	65	35
CAZ	Cephalosporins	Cell wall synthesis	106	57	1
IPM	Carbapenems	Cell wall synthesis	123	40	1
MEM	Carbapenems	Cell wall synthesis	123	39	2
POL	Lipopeptides	Membrane permeability	0	0	164
AMK	Aminoglycosides	Protein synthesis	88	75	1
GEN	Aminoglycosides	Protein synthesis	100	56	8
MIN	Tetracyclines	Protein synthesis	1	158	5
CIP	Fluoroquinolones	DNA synthesis	127	36	1
LEV	Fluoroquinolones	DNA synthesis	88	39	37
SXT	Sulfonamides	Folic acid synthesis	91	73	0

Consistent with publicly available data, the 12 antibiotics used in this study could be categorized into three clusters (Fig. 5C). In terms of prediction accuracy, RF-based models outperformed the rule-based method in group IIb and group III. Additionally, RF-based demonstrated significantly lower rates or MEs and VMEs compared to the rule-based method for all drugs except SXT. Furthermore, the key feature sets of 12 and 11 drugs were found to be significantly enriched with ARGs and IS elements, respectively (*P* ≤ 0.05) (Fig. S7). These results not only confirmed the excellent performance of our model with dependent data but also provided further evidence for the crucial role of IS elements in *A. baumannii* AMR.

**Fig 6:**
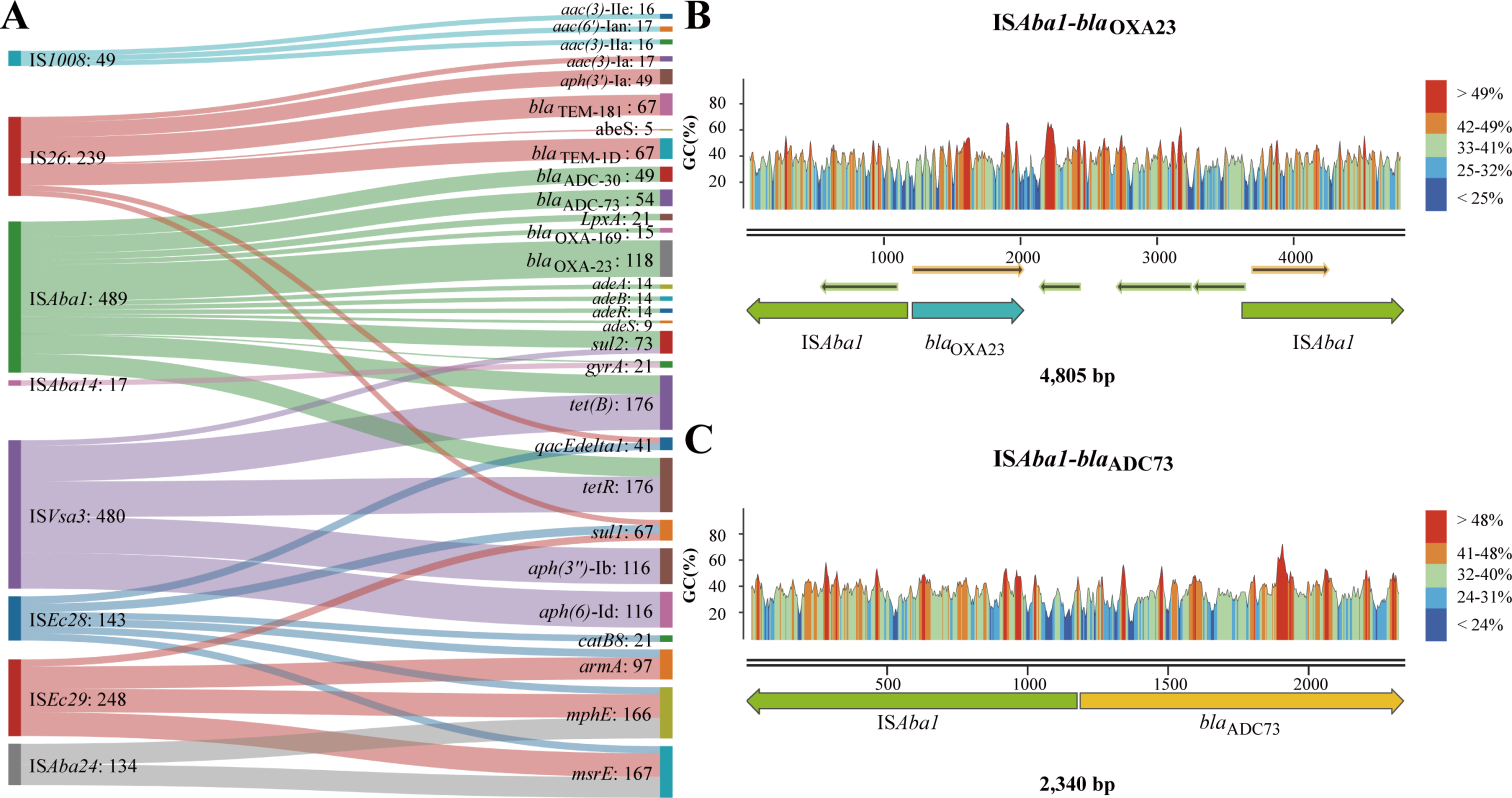
IS-ARG pairs distribution in 164 self-collected *A. baumannii* isolates. (**A**) Sankey diagram showing the prevalence of different IS elements detected closely to the ARGs in clinical isolates. (**B to D**) Genetic arrangement of IS*Aba1* with *bla*_OXA-23_, IS*Aba1* with *bla*_ADC-73_, and IS*Vsa3* with *tetR*, respectively. DNA segments were extracted from corresponding clinical genome sequences and images were initially created using SnapGene.

Based on high-quality genomes of 164 self-collected isolates, a more comprehensive and complete set of IS-ARG pairs was identified (Fig. S8). These isolates were then categorized into five clusters based on the patterns of IS-ARG pairs using bidirectional unsupervised clustering (Fig. S9). Cluster 1 consisted of isolates that did not possess any IS-ARG pairs and demonstrated high sensitivity to all drugs, which aligned with observations from public data. Isolates in cluster 2 exhibited sensitivity only to cephalosporins. Isolates in clusters 3, 4, and 5 displayed resistances to cephalosporins, carbapenems, penicillins, and fluoroquinolones. The AMR patterns of these isolates were consistent with the presence of multiple IS-ARG pairs. Notably, numerous IS-ARG pairs were observed in both the public and validation cohorts, further supporting the importance of these interactions in determining resistance phenotypes (Fig. 6A). For example, IS*Vsa3* tended to be located near ARGs that confer resistance to aminoglycosides, tetracycline, and sulfamides. IS*Aba1* was associated with ARGs responsible for cephalosporin, carbapenem, and tetracycline resistance. Similarly, IS*26* tended to co-occur with genes conferring resistance to aminoglycosides, cephalosporins, and sulfonamides. Interestingly, despite significant differences in isolation time and sampling source between the public and validation isolates, the interaction patterns between IS elements and ARGs remained largely conserved. This suggested that IS elements may interact with ARGs in specific ways to influence the resistance phenotypes of *A. baumannii*.

In addition, the presence of ARG alone was insufficient to cause antibiotic resistance. The key factor for antibiotic resistance was the presence of specific IS-ARG pairs, which were confirmed in the validation data. For example, IS*Aba1-bla*_OXA-23_ and IS*Aba1-bla*_OXA-169_ pairs were identified in 117 and 15 strains, respectively, and directly influenced resistance phenotypes of carbapenems. The distances between IS*Aba1* and *bla*_OXA-23_ were consistent in these strains. In 117 isolates, IS*Aba1* was located 35/36-bp upstream of *bla*_OXA-23_, while in 79 samples, it was found nearly 1,590-bp downstream of it (Fig. 6B). Based on these relationships, the 164 isolates could be divided into three distinct clusters (Fig. S10). In cluster 3, one isolate had *bla*_OXA-23_ without any IS present upstream or downstream of it, and it exhibited sensitivity or intermediate resistance to IMP and MEM. Once IS*Aba1-bla*_OXA-23_ or IS*Aba1-bla*_OXA-169_ pairs were identified, all isolates became resistant to IMP and MEM. Another example demonstrated the association between interactions of IS*Aba1* and *bla*_ADC_ gene and the resistant phenotypes of cephalosporins. IS*Aba1* was simultaneously found in isolates containing cephalosporin resistance-related genes *bla*_ADC-30_ or *bla*_ADC-73_, and it was located 10/11-bp upstream of these two genes (Fig. 6C). The presence of IS*Aba1-bla*_ADC_ pair was the key factor of the cephalosporin resistance phenotype. Some isolates that had *bla*_ADC-2_ and *bla*_ADC-79_ genes without nearly interacting IS elements were sensitive to CAZ and CFE (Fig. S11). These results indicated that the presence of specific IS-ARG pairs corresponded to resistance phenotypes of certain antibiotics in *A. baumannii*.

## DISCUSSION

In this study, the associations between genomic elements and AMR phenotypes were systematically investigated, and the significant implication of IS elements in AMR was reported. A total of 1,012 publicly available isolates and 164 self-collected isolated isolates were analyzed, along with 20 and 14 antibiotic phenotypes, respectively. The performance of the RF, SVM, and LR algorithms in model construction was evaluated and compared. Through this approach, we quantified the contribution of IS elements to AMR and identified interaction patterns of IS elements and ARGs, known as IS-ARG pairs, which were found to be the key predictor for AMR in most antibiotics. Our findings provided candidate biomarkers for AMR phenotype prediction, and functional experimental validation of these IS-ARG pairs is essential for a deeper understanding of their mechanisms of action.

With the rapid accumulation of public genomic data, machine learning has in recent years proven to be an extremely useful tool for studying the AMR phenotypes of clinical pathogens (14). It has demonstrated broad applicability and accuracy across various pathogens, including clinically common ESKAPE bacteria, non-typhoidal *Salmonella*, and *M. tuberculosis* (37–39). In addition to clinical requirements, the growing reports of foodborne MDR strains, such as *Salmonella* (40, 41), suggests that the potential applications could be further expanded. Beyond prediction, machine learning models are also valuable for researching resistance mechanisms, aiding in the identification of resistance genes, mutation sites, and potential resistance determinants (42). In this study, based on the features associated with resistance phenotypes screened using RF-based model, it was found that the key factors for AMR mainly consisted of three types of genetic elements: ARGs, IS elements, and other genetic components. Previous studies have already described the mechanisms of how some IS elements affecting AMR phenotypes, such as IS*Aba1* (29, 30) and IS*26* (35, 36), which validated the effectiveness of our method. Moreover, it is particularly interesting that the interaction of IS elements and ARGs exhibited conserved patterns for most antibiotics in both public and self-collected data. For example, IS*Aba1* was more likely to interact with beta-lactamase resistance genes *bla*_ADC-79_ and *bla*_OXA-23_, while IS*26* was more closely related to aminoglycoside resistance genes, and IS*Vsa3* was more likely to form relationships with aminoglycoside and tetracycline resistance genes. Furthermore, the distances between some ISs and ARGs were found to be conserved, suggesting that the interaction between IS elements and ARGs might be proximity-dependent. Conservation in distance could be due to their roles as components of composite transposons, which exhibit structural conservation, or because IS elements can directly carry and mobilize one or more resistance genes within their adjacent regions. In this study, the association between IS-ARG pairs and resistance phenotypes in *A. baumannii* was confirmed. However, whether these results can be extended to other species requires further investigation with additional data.

Notably, the RF-based models achieved better performance than other methods in terms of phenotypic resistance for most antibiotics. Our results showed that the RF-based models had higher sensitivity and specificity and lower levels of ME and VME. The choice of ARG database had a significant influence on the genotype-phenotype consistency for rule-based method in *A. baumannii* (Fig. 2). The CARD database reported more false-positive results, while the Resfinder database reported false-negative results. When using a rule-based approach to predict AMR, it would be optimal to combine results from multiple databases.

In this study, we enrolled 164 isolated *A. baumannii* strains from a single hospital. Our findings highlighted the importance of complete genomes in identifying a more comprehensive and diverse set of IS-ARG pairs, as their higher accuracy and contiguity enable a more robust analysis. Although we identified many IS-ARG pairs that significantly influence AMR phenotypes, the accuracy of our model could be further enhanced with a broader range of clinically isolated strains from multiple centers and a more diverse spectrum of AMR phenotypes. This would further improve and deepen our understanding of the roles of IS elements in AMR. Additionally, if sufficient evidence establishes clear causal relationships between some IS-ARG pairs and resistance phenotypes, we would be able to develop preclinical diagnostic tools for resistance that meet the critical requirements of high accuracy and low error rates. These tools can efficiently and rapidly detect resistance, providing valuable guidance for the rational use of antibiotics and helping to reduce the emergence and spread of antimicrobial drug resistance in pathogens.

Overall, this study constructed RF-based model for predicting AMR phenotypes using 1,012 publicly available isolates, which demonstrated excellent performance. Additionally, it was shown that the interaction between IS elements and ARGs was a key factor in the multidrug resistance of *A. baumannii*. The interaction patterns between ISs and ARGs were found to be relatively conserved, with these elements occurring in specific combinations and at relatively fixed distances from each other. Most importantly, these findings were validated in an independent data set of 164 self-collected isolates.

## MATERIALS AND METHODS

### Genomic and antibiotic resistance phenotypes data collection

In this study, we used two data sets referred to as the public data and the self-collected data. The public available data were originally obtained from PATRIC (https://patricbrc.org/). A total of 1,012 *A*. *baumannii* isolates from 1999 to 2019 with data of both antimicrobial susceptibility phenotypes and the corresponding genome sequences or whole genome sequencing data were used. AST results of 20 antibiotics were obtained from PATRIC, including beta-lactams (beta-lactam compound: SAM), cephalosporins (CPM, CTX, CAZ, and CRO), carbapenems (DOR, IMP, and MEM), aminoglycosides (AMK, GEN, and TOB), fluoroquinolones (CIP, LEV, and MXF), lipopeptides (COL and POL), tetracyclines (MIN, TGC, and tetracycline [TET]), and SXT. The genome sequences of 863 strains were acquired from the National Center for Biotechnology Information (NCBI) (https://www.ncbi.nlm.nih.gov/), and raw whole genome sequencing reads of the remaining 149 strains were downloaded from Sequence Read Archive (SRA; https://www.ncbi.nlm.nih.gov/sra). The public data used in this study were summarized in Table S1.

Furthermore, the 164 clinically isolated strains were collected from the Chinese PLA General Hospital between 2018 and 2020. ASTs corresponding to 14 antibiotics, including beta-lactams (SAM, CPM, CAZ, IPM, MEM, piperacillin [PIP], PIP-tazobactam [TZP]), aminoglycosides (AMK and GEN), fluoroquinolones (CIP and LEV), lipopeptides (POL), MIN, and sulfonamides (SXT) were performed by standard broth microdilution assays. Antibiotic abbreviations and clinical breakpoints were adopted from the Clinical and Laboratory Standards Institute M100 32nd Edition and minimum inhibitory concentrations were then converted to susceptible (S), resistant (R) or intermediate (I) determinations.

### Illumina sequencing

Genomic DNAs of 164 clinical isolates were extracted with the SDS method using QIAamp DNA Mini Kit (Qiagen, Hiden, Germany) and quantified by Qubit 2.0 Fluorometer (Thermo Fisher Scientific, Waltham, MA, USA). The extracted DNAs were fragmented to a size of 350 bp by sonication, and sequencing libraries were prepared using NEBNext Ultra DNA Library Prep Kit for Illumina (NEB, Ipswich, USA). Paired-end sequencing with 150 bp was conducted on the Illumina NovaSeq 6000 platform (Illumina Inc., San Diego, CA, USA), with average sequencing depth of 652× (Table S7).

### Nanopore sequencing

Genomic DNA of each clinically isolated strain was extracted for nanopore sequencing using the ZymoBIOMICS DNA Miniprep Kit D4300 (Zymo Research, Irvine, CA, USA) following the manufacturer’s recommendations. Sequencing libraries were prepared using ONT’s 1D ligation sequencing kit (SQK-LSK109) with native barcoding (EXP-NBD196), according to the manufacturer’s instructions. The final sequencing libraries were sequenced using R9.4.1 flowcells on a GridION X5 platform, with average sequencing depth of 115× (Table S8).

### Sequencing data processing

The SRR files, corresponding to available isolates in PATRIC, were downloaded from NCBI SRA database and subsequently converted to fastq format by sratoolkit (43) (fastq-dump --split-3). Quality control processes including adapter trimming, removal of low-quality reads, and elimination of short reads were carried out by Trimmomatic (44). The base calling of fast5 files generated by nanopore sequencing was performed using Guppy v3.0.5 (45) (flip-flop HAC model). Low-quality and short nanopore reads were filtered with parameters of “sequence quality >8” and “sequence length >1000 bp” using NanoFilt v2.8.0 (46).

### Genome assembly and annotation

Unicycler v0.4.8 (47) was utilized to assemble the genomes of the public PATRIC isolates with the following parameters: --min_fasta_length 500, -t 20, --keep 2. Multiple assemblers, including Unicycler v0.4.8 (--min_fasta_length 500, -t 20, --keep 2), Canu v2.1.1 (48) (genomeSize = 4 m, corOutCoverage = 40), Flye v2.8 (49) (--nano-corr, --genome-size 4 m, --threads 8, --iterations 1), and wtdbg2 v2.5 (50) (-t 8, -x corrected, -g 4 m), were used for the genome assembly of long reads obtained from 164 self-collected clinical isolates. For each strain, assembled sequence with the highest N50 value was initially polished by Racon v1.4.20 (51) (-u, -t 8) using the nanopore long reads, followed by three rounds of polishing with Pilon v1.23 (52) (--changes, --fix all) using the high-quality Illumina pair-end short reads. Then, the polished sequences were circularized using Circlator v1.5.5 (53) with default parameters. The ultimately obtained genome sequences were assessed with BUSCO v5.2.1 (54) (-m genome option) using the bacteria library of odb10 (*n* = 124). The genomes were annotated by Prokka v1.13.7 (55) with default parameters.

### Identification of IS elements, ARGs, and IS-ARG pairs

The IS elements and ARGs were identified by querying the genome sequences against IS finder (56), CARD v3.1.0 (11), and Resfinder (12) (version 13 April 2021) databases using Abricate v0.8 (https://github.com/tseemann/abricate) with the strict thresholds of identity ≥90% and coverage ≥90%. The ARGs identified by the CARD and Resfinder databases were consolidated into a non-redundant gene set, retaining sequences identified from both databases with a high degree of similarity (identity ≥ 90% and coverage ≥ 90%). An IS-ARG pair was identified based on two criteria. First, the IS and its associated ARG must be located on the same sequence. Second, the base-pair distance between them should not exceed 5 kb, which is an empirically determined threshold based on previous study (57). Only IS-ARG pairs exhibiting consistent directionality (upstream or downstream) and possessing a distance variation between ARG and IS not exceeding 10 base pairs were regarded as identical.

### Feature selection and model construction

To improve the predictive performance of AMR phenotypes, we developed phenotypic prediction model for *A. baumannii* using the RF algorithm (Fig. S2). Three different types of elements constituted the feature inputs (F): (i) a matrix of presence or absence of genes annotated by Prokka, (ii) a matrix of IS elements, and (iii) a matrix of ARGs after removing redundant results between the CARD and Resfinder databases. For the features defined above, F_ij_ is 1 if feature i is present in isolate j and 0 otherwise.

“RandomForestClassifier” function in Scikit-learn Python package (58) (www.scikit-learn.org) was employed to select important feature sets and construct the phenotypic prediction model. Firstly, drugs were discarded if the number of isolates with resistant or sensitive phenotypes was less than 5, to ensure a sufficient number of samples in each class for effective model training. Secondly, 70% of the isolates were randomly sub-sampled as the training set, while the remaining were designated as the validation set, to avoid bias that could arise from manually selecting the data. The significance of each feature in the training set was evaluated and ranked using the “rank” function in Pandas Python package (59) with the “dense” method. Features with comparable importance were grouped together, and the groups with higher importance were assigned higher ranks, reflecting their top significance. Thirdly, the processes of randomly sub-sampling and feature importance calculation were repeated 1,000 times, and the mean dense rank of each feature was obtained. This repetition ensured that the results were robust and minimized the risk of false positives by averaging the dense ranks over multiple iterations. Features with mean dense rank smaller than 100 were selected as the key feature sets and used to train the phenotypic prediction model, representing approximately the top 10% of features after 1,000 iterations, ensuring that only the most significant features are used.

### Indexes for model evaluation

After feature selection, in-house RF-based algorithm was employed for model construction, along with SVM and LR. The model was evaluated in the validation set using accuracy (ACC), MCC, VME, ME, and F1 score, which is a weighted harmonic mean of recall and precision. The calculation formulas or definitions are described as follows:


(1) $$ACC=\frac{TP+TN}{TP+FP+TN+FN}$$

where TP denotes true positive, TN denotes true negative, FP denotes false positive, and FN denotes false negative. Positive and negative corresponded to the susceptibility label R and S.


(2) $$MCC=\frac{TP*TN-FP*FN}{\sqrt{\left(TP+FP\right)\left(TP+FN\right)\left(TN+FP\right)\left(TN+FN\right)}}$$

where TP, TN, FP, FN are the same as in equation 1. As a reference performance measure, MCC is more stringent in unbalanced data sets, ranging from −1 to 1 (−1 represents total disagreements between observed and predicted values, while 1 represents perfect agreement).


(3) $$F1=\frac{2*P*R}{P+R}$$

(4) $$P=\frac{TP}{TP+FP},\;R=\frac{TP}{TP+FN}$$

where P is precision and R is recall in equation 3.

VME represents the fraction of resistant samples that are predicated to be susceptible, and ME is defined as the proportion of sensitive samples that are predicated to be resistant. The process of model evaluation was also repeated 1,000 times. Ultimately, the average values of these metrics were used to evaluate performance of models.

### Phenotypic prediction using rule-based method

Rule-based method predicts antibiotic resistance through detecting the presence of one or more known resistance genes or mutations. For each ARG related with certain antibiotics, the number of sensitive and resistant isolates containing it was counted and compared using Fisher’s exact test. Generally, ARGs existing in resistant isolates far more than that in sensitive were more likely to be considered as key factors for antibiotic resistance in the rule-based method.

### Statistical analyses

All statistical analyses were conducted using R software v4.1.0 (60). Differences were considered as statistically significant when *P* ≤ 0.05.

## Data Availability

The sample information, raw data, and assembled genome sequences generated in this study were uploaded to National Genomics Data Center (NGDC) (https://ngdc.cncb.ac.cn/) with the project number PRJCA009915. The sequenced NGS reads and nanopore reads were submitted to GSA database of NGDC under accession number CRA007130. The public data used in this study were described in the supplemental data. The code related to the RF model used in this study can be assessed on GitHub (https://github.com/YHWen-bio/AMR_Predict).

## SUPPLEMENTAL MATERIAL

Supplemental Figures mbio.02852-24-s0001.docx Figures S1 to S11.

Table S1 mbio.02852-24-s0002.csv Genome information and antibiotic resistance phenotype of 1,012 public *A. baumannii* isolates.

Table S2 mbio.02852-24-s0003.csv Performance of phenotypic prediction models in 1,012 public *A. baumannii* isolates.

Table S3 mbio.02852-24-s0004.csv The statistics of interactions between ISs and ARGs in 1,012 public *A. baumannii* isolates.

Table S4 mbio.02852-24-s0005.csv The antimicrobial susceptibility testing results of 164 self-collected *A. baumannii* isolates.

Table S5 mbio.02852-24-s0006.csv The statistics of genome qulities of 164 self-collected *A. baumannii* isolates.

Table S6 mbio.02852-24-s0007.csv Performance of phenotypic prediction models in 164 self-collected *A. baumannii* isolates.

Table S7 mbio.02852-24-s0008.csv Statistics of Illunima short reads of 164 self-collected *A. baumannii* isolates.

Table S8 mbio.02852-24-s0009.csv Statistics of Nanopore long reads of the 164 self-collected *A. baumannii* isolates.

ASM does not own the copyrights to Supplemental Material that may be linked to, or accessed through, an article. The authors have granted ASM a non-exclusive, world-wide license to publish the Supplemental Material files. Please contact the corresponding author directly for reuse.
