# Supplementary material for: Large-scale genomic analysis reveals significant role of insertion sequences in antimicrobial resistance of Acinetobacter baumannii
Source: mBio. 2025 Feb 20;16(3):e02852-24. doi: 10.1128/mbio.02852-24 (PMC11898611; doi:10.1128/mbio.02852-24)
Supplement: Supplemental Figures — Figures S1 to S11. [file mbio.02852-24-s0001.docx]

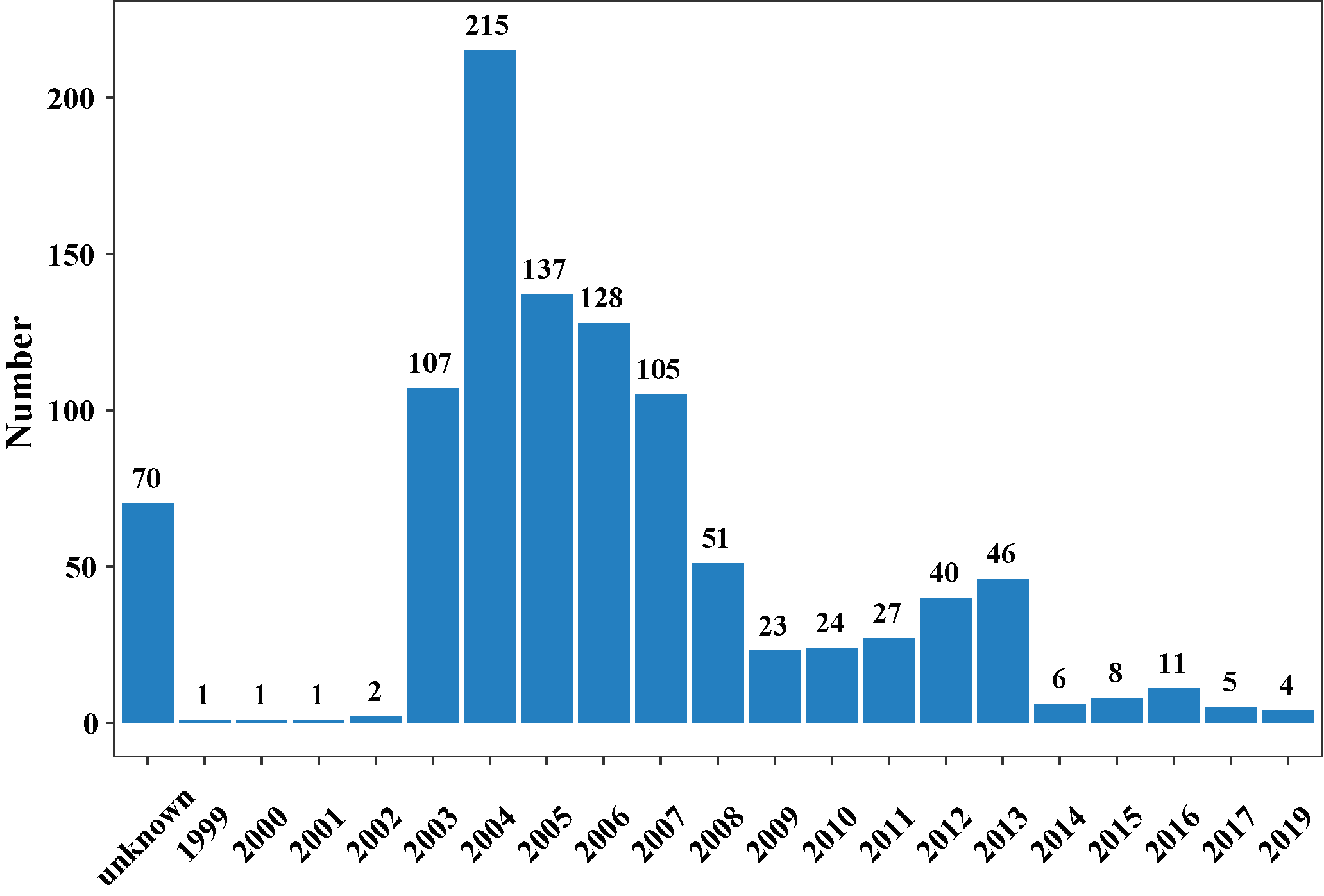


**Fig S1** Barplot of the public samples’ number collected from different years.


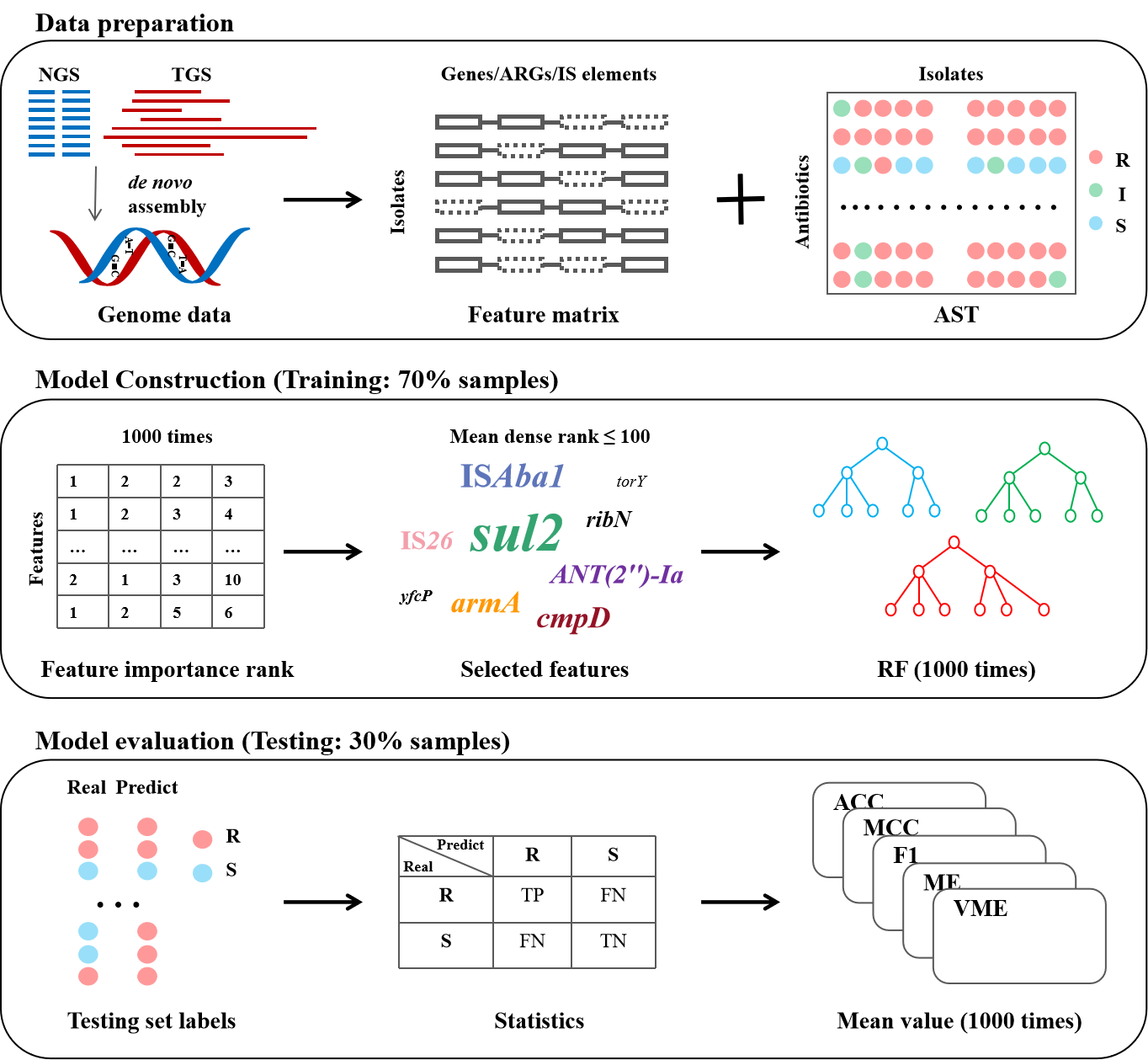


**Fig S2** Workflow of Random Forest-based phenotypic prediction framework. Genome sequence data were obtained by *de novo* assembly using short reads or long reads. Feature matrix containing genes, IS elements, ARGs was built by sequence alignment. Randomly sampling 70% of the datasets into training set, and the remaining datasets were regarded as testing set. 1000 different training-testing pairs were produced by random splitting. Features were selected by the mean dense feature importance rank in 1000 RF models training by training sets. For each training-testing pair, the model was trained by training set and evaluated by training set.


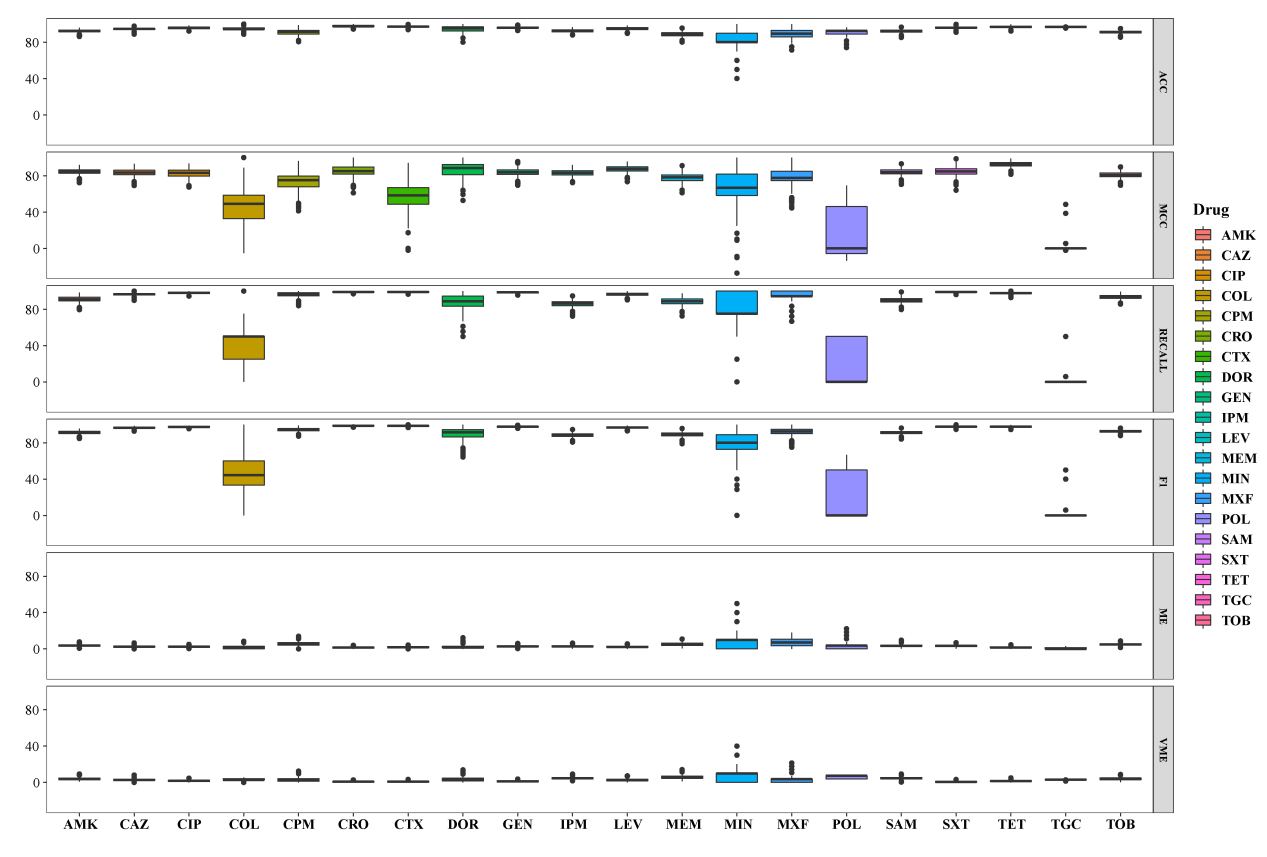


**Fig S3** Performance of RF-based models based on 1000 random splits of public data. Boxplots represent distribution of ACC, MCC, F1, ME, VME for the corresponding testing datasets.


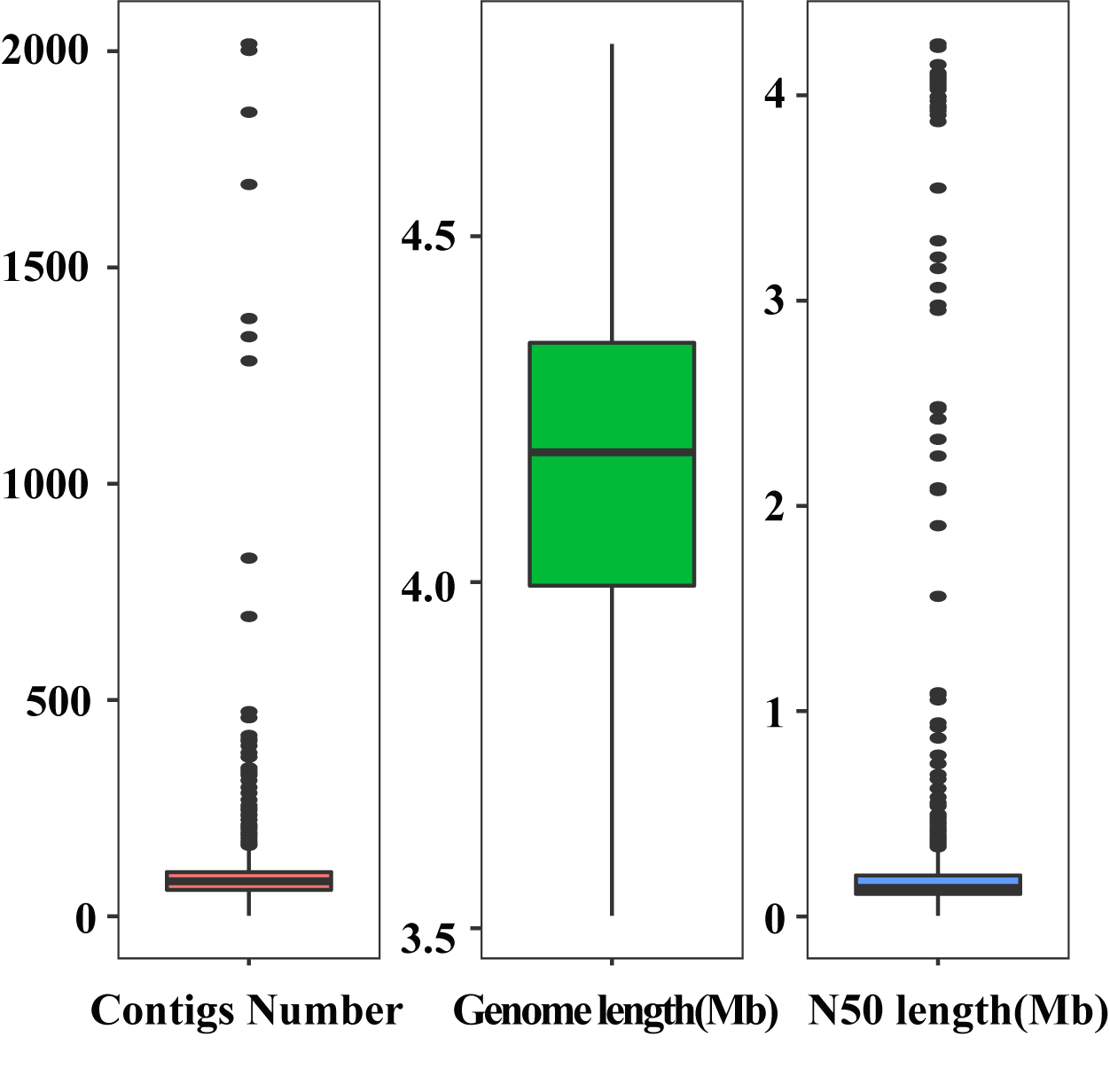


**Fig S4** Assembly metrics of 1,012 publicly sourced *A. baumannii* genomes. Box plots represent the distribution of the number of contigs, total length, and N50 for these 1,012 genomes.

**
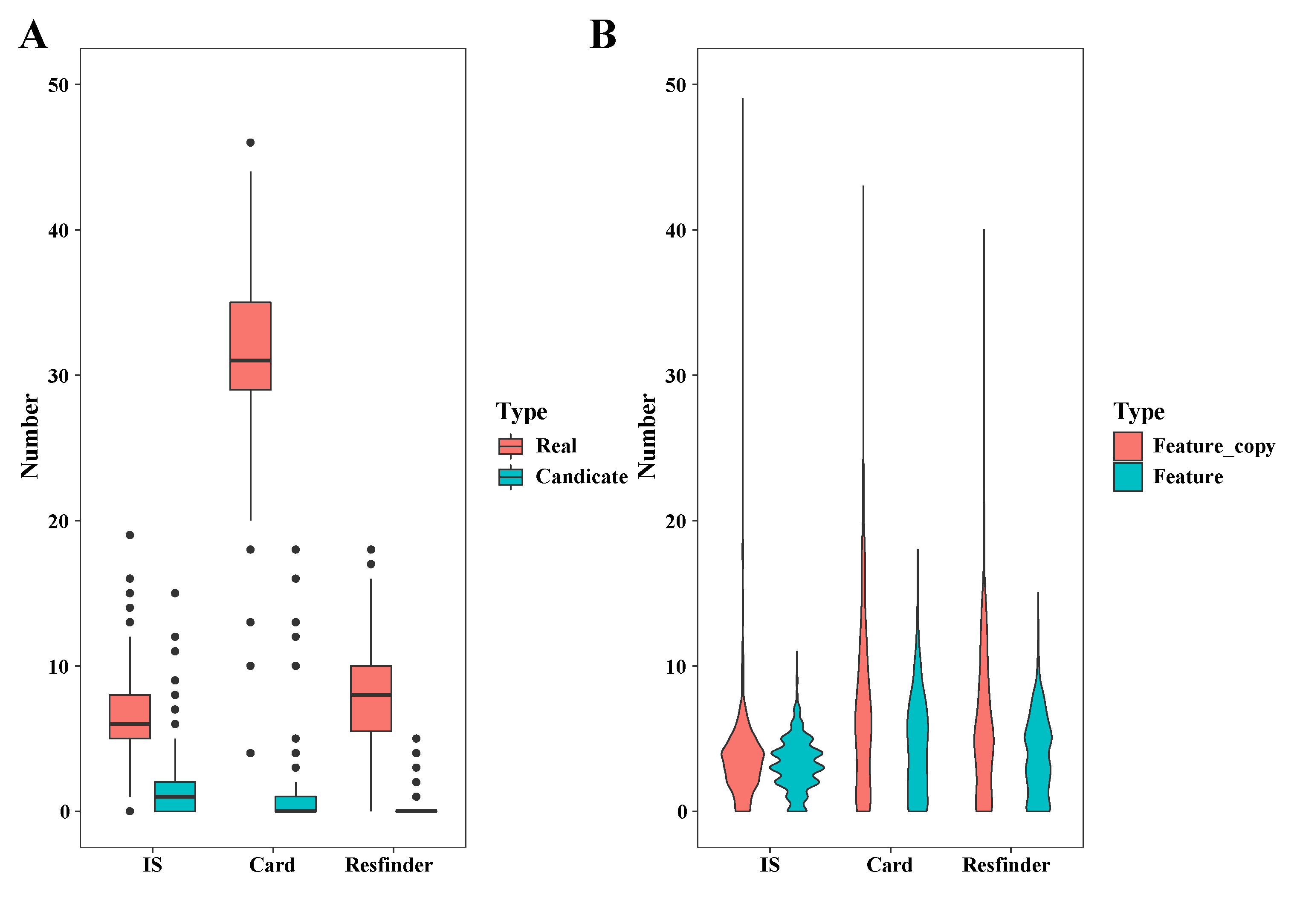
Fig S5** Boxplots of candidate features and margin features in public genomes. A) Real features were identified by method in Materials and Methods. Candidate features were defined as the ARGs or IS elements located within 100bp of the genome contig margin with a minimum consistency of 90 and coverage below 90. Features of which have already been recognized as real features in other location would be filtered. B) The distribution of margin feature number and feature copy. Margin features were defined as the real features of which located within 1000bp of the genome contig margin. The IS-ARG pairs related with margin features may be lost.


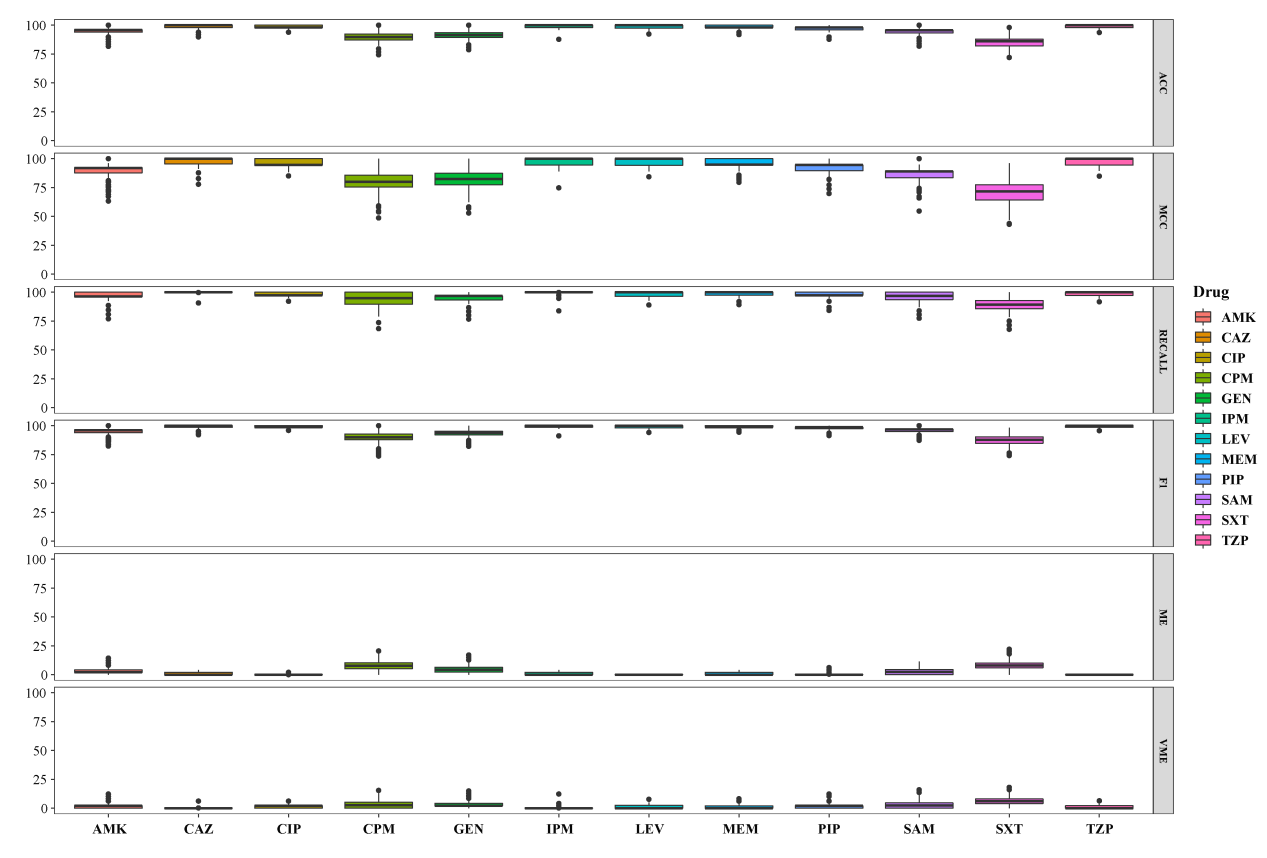
**Fig S6** Performance of RF models based on 1000 random splits of clinical data.


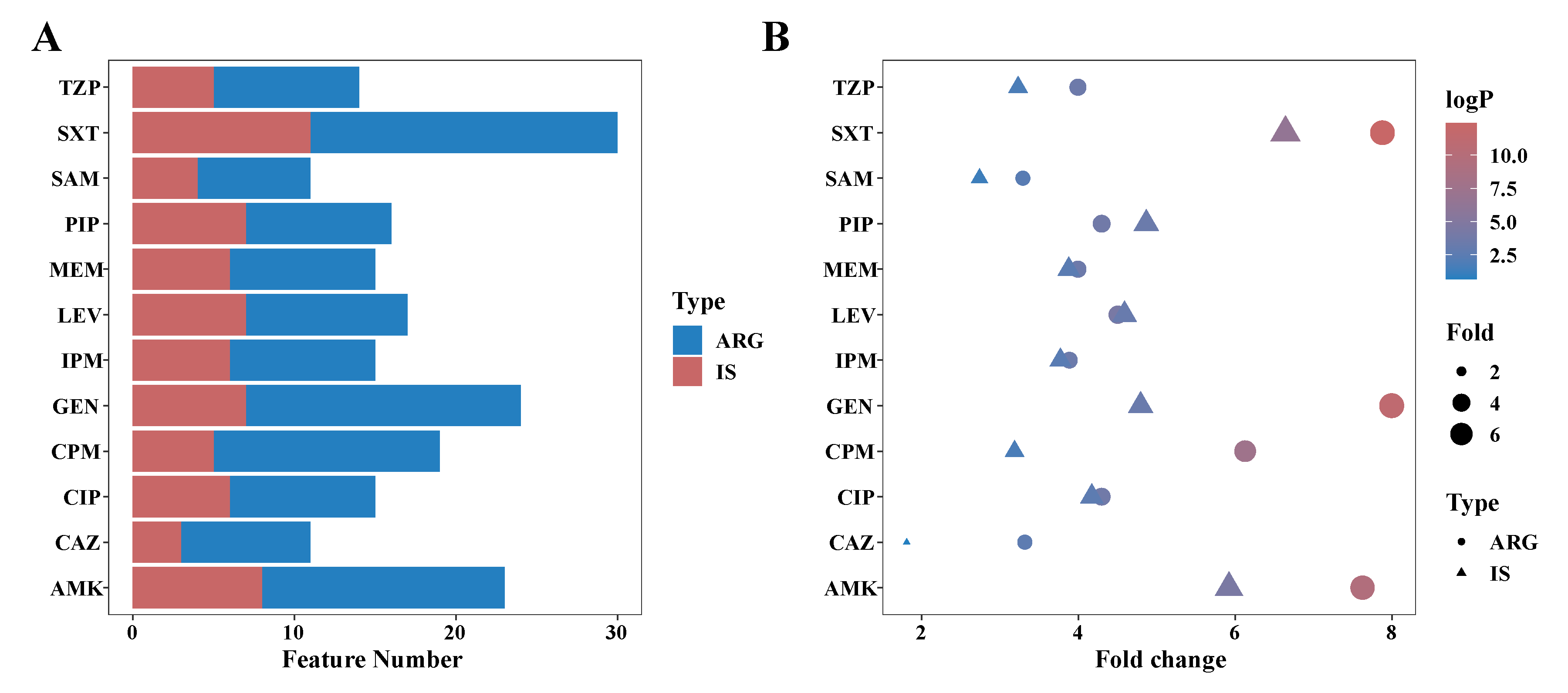


**Fig S7** Selected ARG and IS features for 12 drugs by RF-based models. Left is the features number of IS elements and ARGs, colored according to feature type. Right is the enrichment analysis of selected features (ARGs and IS elements) by hypergeometric testing in different drugs.


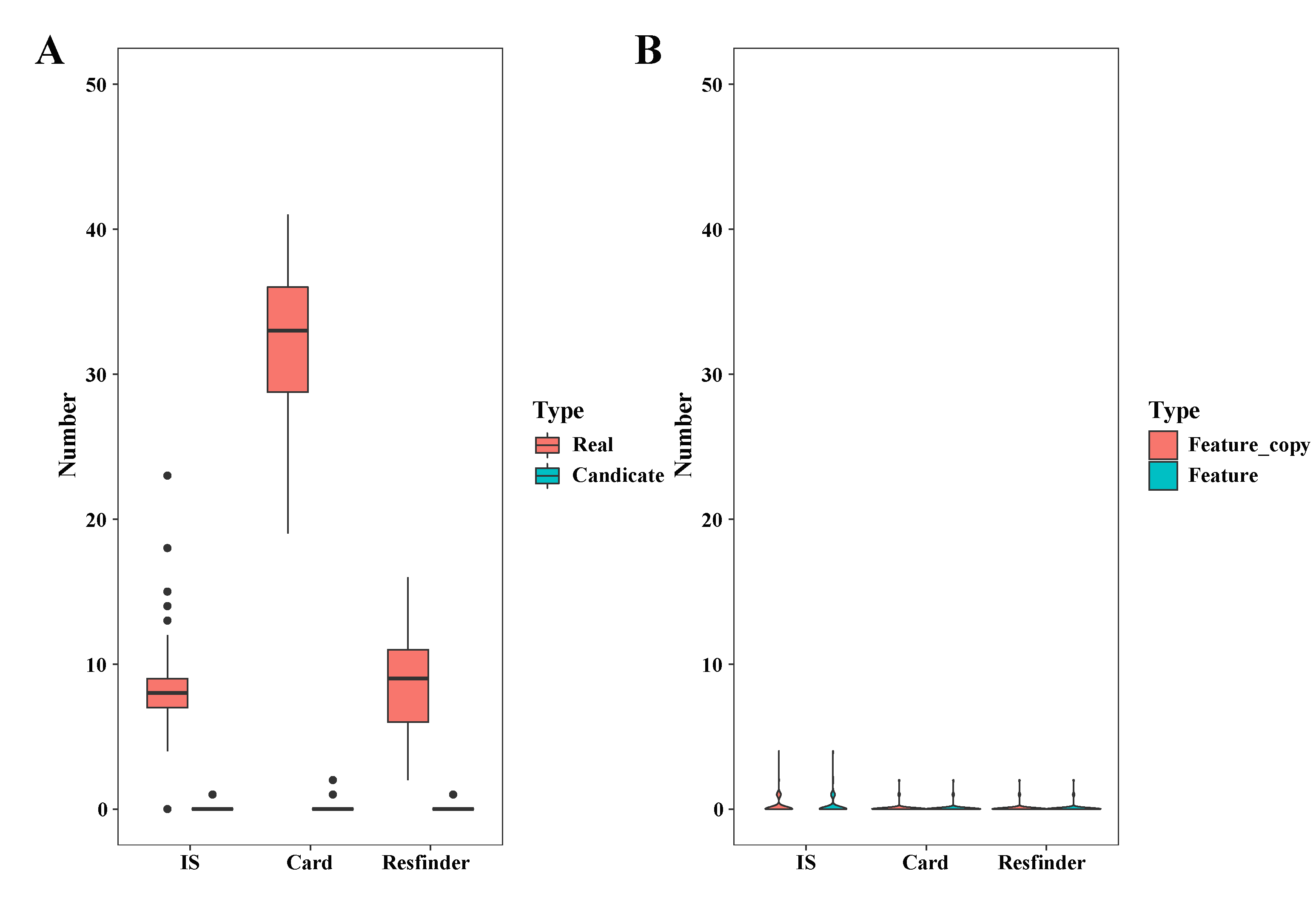


**Fig S8** Boxplots of candidate features and margin features in clinical genomes assembled by NGS short reads and Nanopore long reads.


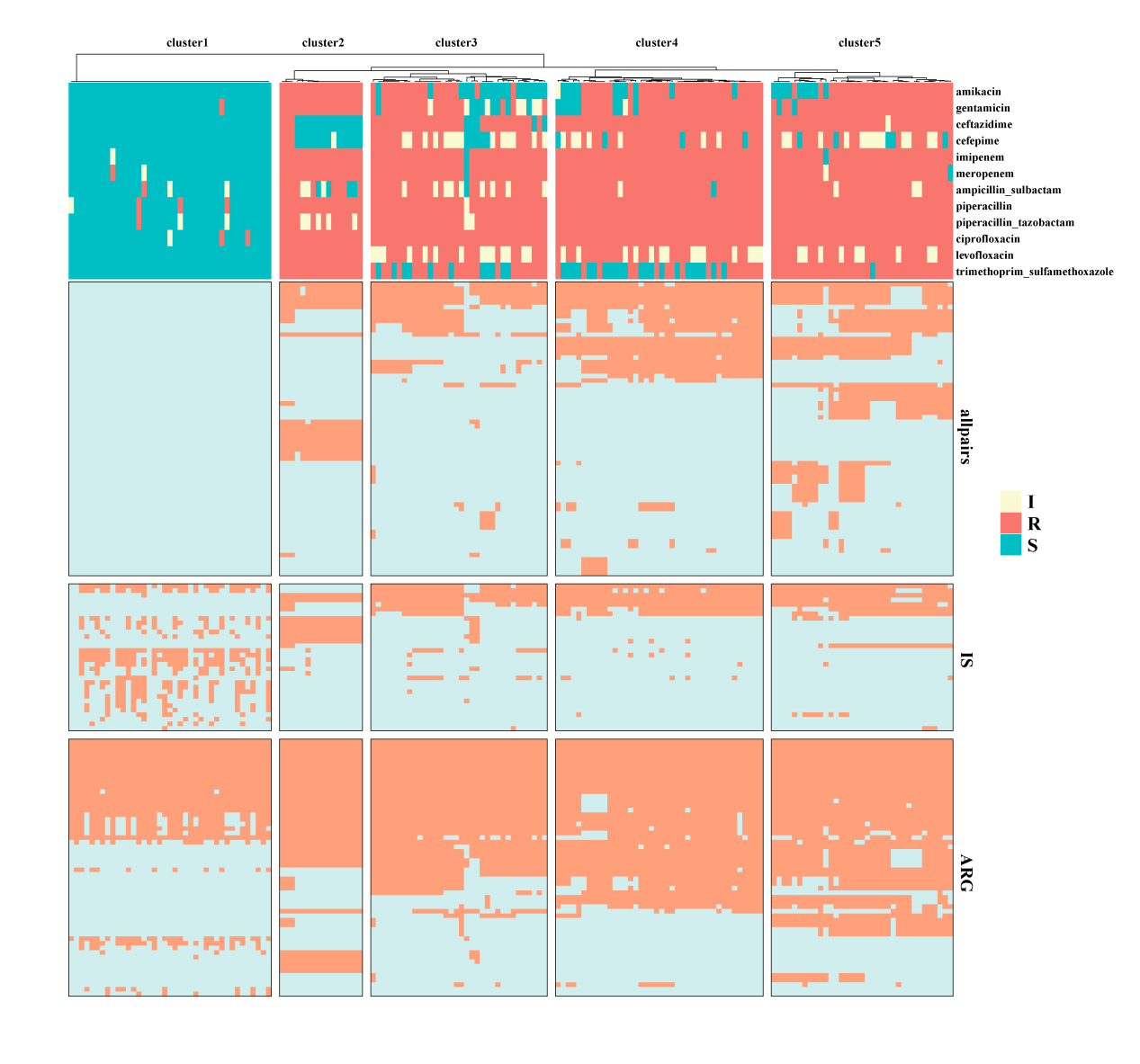


**Fig S9** ARGs, IS elements and IS-ARG pairs distribution in 164 clinically isolated data.


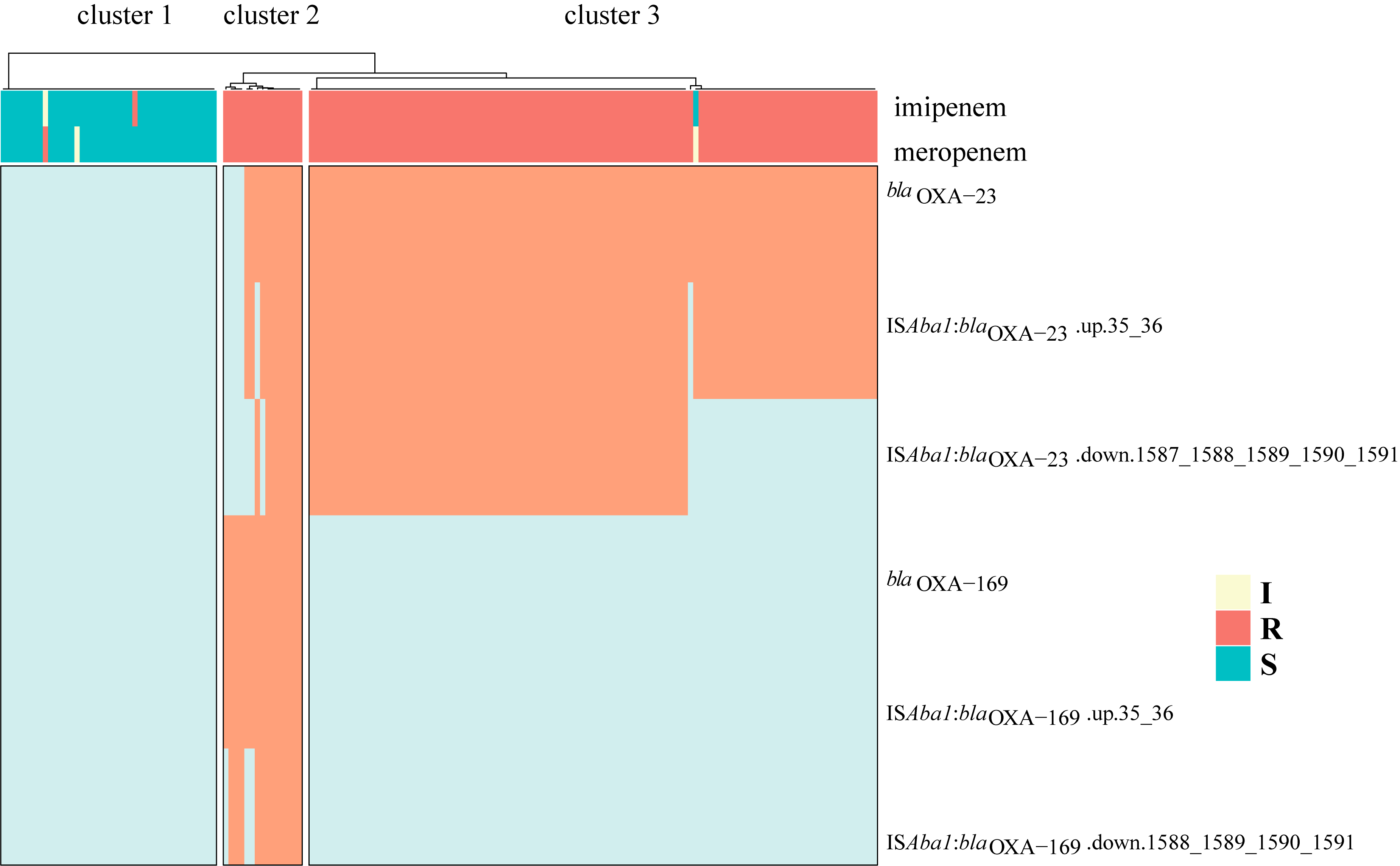


**Fig S10** Detailed detecting of *bla*_OXA-23_, *bla*_OXA-169_ and corresponding IS-ARG pairs in 164 clinically isolated samples. Unsupervised bidirectional clustering was performed based on these features and samples were divided into three clusters. The top bars above the heatmap show the antibiotics sensitivity for IMP and MEM, respectively.

**
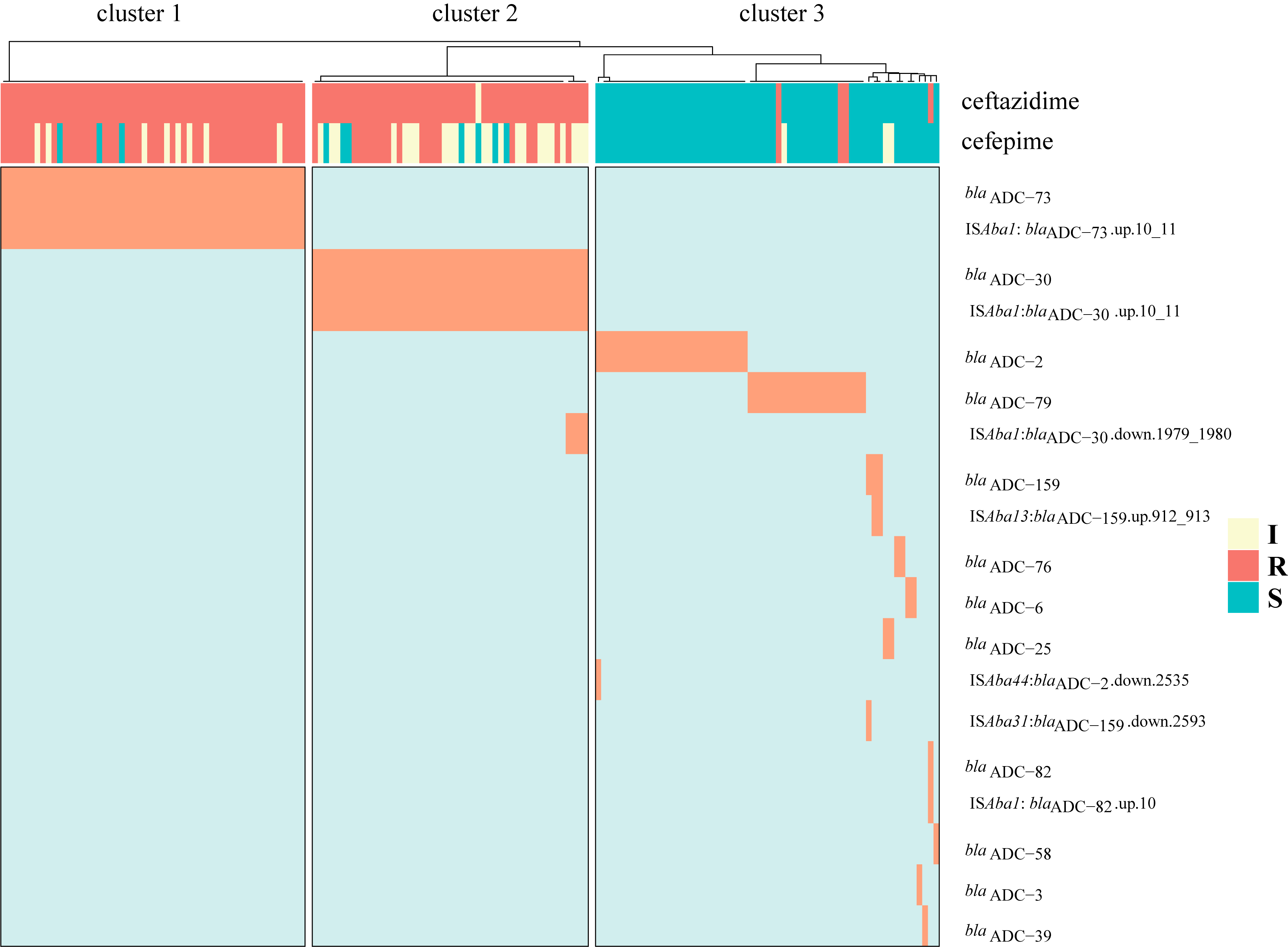
**

**Fig S11** Detailed detecting of cephalosporinase (*bla*_ADC_) and *bla*_ADC_ related IS-ARG pairs in 164 clinically isolated samples. The top bars above the heatmap show the antibiotics sensitivity for CAZ and CPM, respectively.
